# Wild food plants and fungi used in the mycophilous Tibetan community of Zhagana (Tewo County, Gansu, China)

**DOI:** 10.1186/s13002-016-0094-y

**Published:** 2016-06-01

**Authors:** Jin Kang, Yongxiang Kang, Xiaolian Ji, Quanping Guo, Guillaume Jacques, Marcin Pietras, Nasim Łuczaj, Dengwu Li, Łukasz Łuczaj

**Affiliations:** College of Forestry, Northwest A&F University, Yangling, Shaanxi 712100 People’s Republic of China; Yangling Vocational & Technical College, Yangling, Shaanxi 712100 People’s Republic of China; Forestry Academy of Bailongjiang Forestry Administration Bureau, Liangshui, Gansu 746010 People’s Republic of China; Department of Plant Taxonomy and Nature Conservation, University of Gdansk, Wita Stwosza 59, Gdańsk, 80-308 Poland; Institute of Dendrology Polish Academy of Sciences, ul Parkowa 5, Kórnik, 62-035 Poland; French National Centre for Scientific Research, Centre de recherches linguistiques sur l’Asie orientale, 2 rue de Lille, Paris, 75007 France; University of Glasgow (Bachelor of Art degree course), Glasgow, UK; Department of Botany, Institute of Applied Biotechnology and Basic Sciences, University of Rzeszów, Werynia 502, Kolbuszowa, 36-100 Poland

**Keywords:** Wild edible plants, Diebu (Tewo), Thebo, Ethnobotany, Ethnomycology, Edible mushrooms

## Abstract

**Background:**

The aim of the study was to investigate knowledge and use of wild food plants and fungi in a highland valley in the Gannan Tibetan Autonomous Region on the north-eastern edges of the Tibetan Plateau.

**Methods:**

Field research was carried out in four neighbouring villages in a mountain valley of the Diebu (Tewo) county, surrounded by spruce forests. The study consisted of 30 interviews with single informants, or group interviews (altogether 63 informants). Apart from collecting voucher specimens, we also identified fungi using DNA barcoding.

**Results:**

We recorded the use of 54 species of vascular plants. We also recorded the use of 22 mushroom taxa, which made up the largest category of wild foods. Fruits formed the largest category of food plants, with 21 species, larger than the wild greens category, which consisted of 20 species eaten after boiling or frying and 7 as raw snacks. We also recorded the alimentary use of 10 species of edible flowers and 3 species with underground edible organs. On average, 20.8 edible taxa were listed per interview (median – 21). The most listed category of wild foods was green vegetables (mean – 7.5 species, median – 8 species), but fruits and mushrooms were listed nearly as frequently (mean – 6.3, median – 6 and mean – 5.8, − median 6 respectively). Other category lists were very short, e.g., flowers (mean – 1.3, median – 1) and underground edible parts (mean – 0.7, median – 1).

Wild vegetables are usually boiled and/or fried and served as side-dishes, or their green parts are eaten as snacks during mountain treks (e.g., peeled rhubarb shoots). Wild fruits are mainly collected by children and eaten raw, they are not stored for further use. The most widely used wild staple foods are *Potetilla anserina* roots, an important ceremonial food served on such occasions as New Year or at funerals. They are boiled and served with sugar and butter. The most important famine plants remembered by people are the aerial bulbils of *Persicaria vivipara*. Flowers are used as children’s snacks – their nectar is sucked.

**Conclusions:**

The number of wild taxa eaten in the studied valley is similar to that of other Tibetan areas. The structure of wild food plant taxa is also very typical for Tibetan speaking areas (e.g., the use of rhubarb shoots, *Potentilla anserina, Persicaria vivipara*). The studied community show a high level of mycophilia.

## Background

The Tibetan speaking population inhabits highland areas of Central Asia in five countries of the region, namely China, India, Bhutan, Nepal and Pakistan. Most Tibetans live in the People’s Republic of China. Due to the inaccessibility of many Tibetan-inhabited areas and the political isolation of the region throughout the 20^th^ century, the number of ethnobotanical studies among Tibetans has been disproportionally small relative to the biocultural diversity present in the Tibetan Plateau and adjacent areas [1–7].

So far the wild food plants used by Tibetans have been documented in only a few studies. A team of scholars from Beijing recorded them in the Shangri-La region in Yunnan [2], Boesi researched Litang, Sichuan [1], and Kang and colleagues the Zhouqu county in Gansu [3]. We know even less about the edible fungi used by Tibetans, though existing publications show that in some communities at least a few taxa are widely collected and eaten [1, 3, 4, 8, 9].

One of the least ethnobotanically explored parts of China is the province of Gansu, which lies in the north-eastern part of the range of Tibetan languages and dialects. In previous expeditions some of the authors of this paper explored another area of western-central China, namely two valleys on the northern slope of the Qinling Mountains in Shaanxi [10, 11], and one mountain valley in south-western Gansu inhabited by Tibetans [3]. Our previous study among the Tibetan people was performed in very unusual surroundings for this ethnic group, i.e., a relatively low elevation of around two thousand m a.s.l. In contrast, the study whose results we present in this article concerns a population of Gansu Tibetans living at higher altitudes (around 3000 m a.s.l.), among highland spruce forests.

The documentation of traditional wild foods is important for an understanding of traditional food systems [12–17] and for the cultural heritage of minorities living in China, as rapid economic progress increases food availability, and many lesser-used wild vegetables are becoming forgotten. Nowadays we still can interview many people who have good expertise on the emergency foods used during the 1959–61 famine (see e.g., [10, 11]). These people are 60–80 years old now, so in a few years there will be less of them and those remaining may be too old to go to the forest and show these foods. Eastern Asia is a particularly interesting region for the study of wild foods since nowhere in the world are such large numbers of species of wild vegetables used as in China, Thailand, Japan, Korea and neighbouring countries [17–24]. Tibetans living at high elevations, in areas with species-poor floras, do not use as many species of wild vegetables, but their skill in utilizing local food resources is remarkable and worth documenting.

Tibetans are classified in China as one minority (Z*angzu*). However a mosaic of several Tibetan languages and dialects exists. In the study area Thebo (Diebu) Tibetan is spoken, which is not mutually intelligible with any of the neighbouring varieties, neither Amdo Tibetan, Chone (Zhuoni) nor Mbrugchu (Zhouqu). Few reliable sources exist on this dialect [25].

To fill the gap in the ethnobotanical exploration of the north-western part of China we aimed at documenting the use of wild food plants in one Tibetan valley in SW Gansu.

## Methods

### Study site

**Fig. 1:**
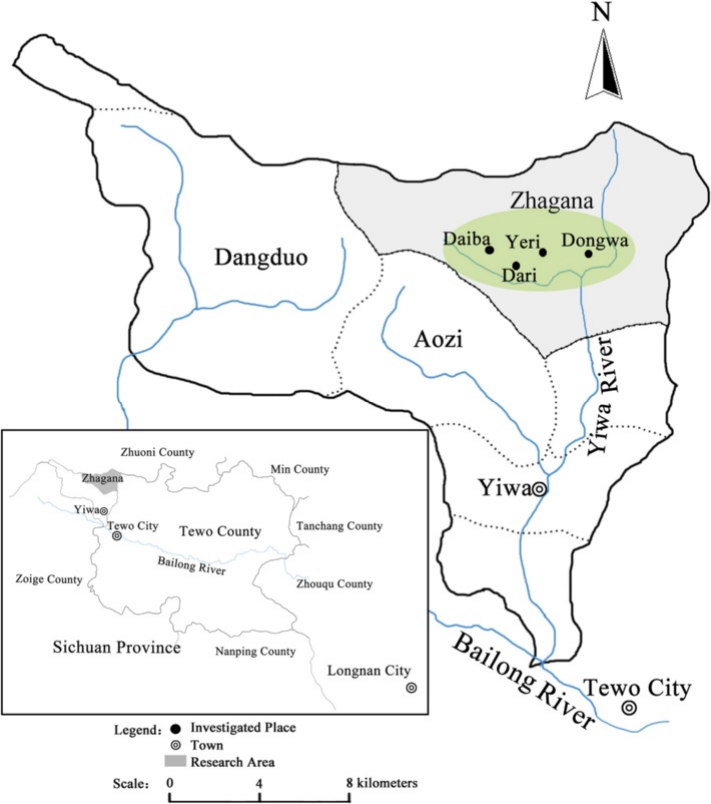
The map of the studied area

The province of Gansu in northwest China (Fig. 1.) has very diverse vegetation. It changes from desert in the north and centre through dry grasslands to deciduous forests in the mountainous south. In the south-west of Gansu, inhabited mainly by Tibetans, the Gannan Tibetan Autonomous Region was established.

Tibetans in Gannan constitute a very diverse collection of subsistence economies and speak a variety of dialects/languages, still not sufficiently explored linguistically. In the northern part of the territory, in the grasslands, animal herders predominate. The Diebu (Tebo, Tewo) Tibetans, although living among forests, live both from animal husbandry and farming, whereas in the southern part of the region (Zhouqu), at lower elevations, plant cultivation is the main source of subsistence.

The studied valley is located along a small river valley (the river Yiwa), which is a tributary of Bailongjiiang (the White Dragon River). Bailongjiang valley and its surrounding areas constitute a mountainous forefront of the Tibetan Plateau. The dominant vegetation in the study area is forest, composed of *Picea crassifolia* Kom., *Picea asperata* Mast., *Picea wilsonii* Mast. *Sabina tibetica* Kom., *Sabina saltuaria* (Rehd. et Wils.) Cheng et W. T. Wang, *Betula platyphylla* Suk.etc. Other frequent types of vegetation are grassland and scrub (*Beberis, Ribes, Rosa, Clematis* and *Rubus*).

**Fig. 2:**
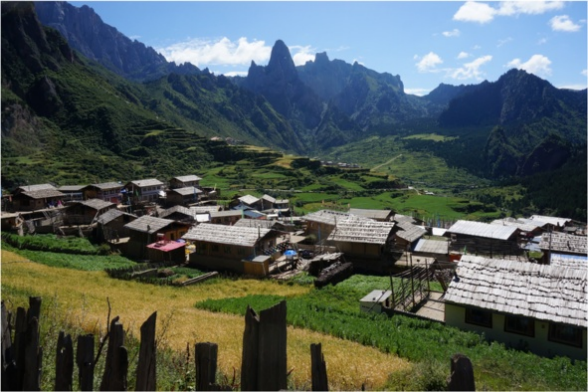
A general view of Zhagana valley

We studied Tibetan villages called Zhagana, near the source of Yiwa River, which is part of the township (*zhen*) of Yiwa, famous for its picturesque landscape (Figs. 1 and 2). These are: Daiba, Yeri, Dari and Dongwa (with a range of coordinates from: N 34°14ʹ13″, E 103°09ʹ54″ to N34 °14ʹ14″, E 103 °10ʹ 58″).

The Yiwa Township covers an area of 365 km^2^. The average annual rainfall is 447–762 mm (mainly in summer months), the annual average temperature is 5.7 °C (at altitude 2500 m a.s.l.) and 2.8 °C (at altitude 3000 m), and the frost-free period lasts on average 142 days per year [26]. The studied villages are purely Tibetan. The altitude at which the houses are located ranged from 2800 to 3300 m a.s.l.

**Fig. 3:**
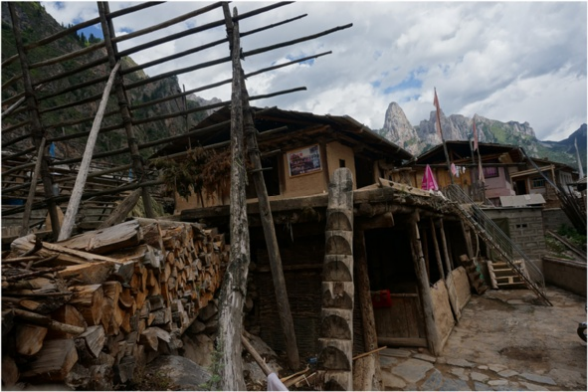
The homestead where Joseph Rock once stayed

The studied population consists of subsistence farmers cultivating oats, barley and potatoes and keeping some yaks, cattle and pigs. Tourism has become an important part of the local economy as the valley has been voted one of the most picturesque places in China. This is causing a suddenly increasing influx of tourists (mainly from the capital of Gansu, Lanzhou). Over the last three years the local population have shifted their main activity from farming to building ad-hoc hiking shelters near many of the village homesteads. Apart from the pristine beautiful landscape, tourists are attracted to the fact that the village of Daiba was one of the headquarters of the famous American botanist explorer Joseph Rock who came to live there in 1925 or 1926 during his expedition to Tewo country (the house where he lived is marked and advertised as a tourist attraction – Fig. 3). Unfortunately Rock’s materials from Gansu sunk with the ship which carried them from Calcutta to the USA during World War II [27–29].

### Data collection

The field research was conducted in August 2015 using the Rapid Rural Appraisal approach [30, 31], and included 30 freelisting interviews (14 interviews – with 11 men and 3 women as single informants and 16 group interviews), which altogether involved 63 people (43 men and 22 women). The mean age of the participants was 55 (median 49, aged from 21 to 83). The research was carried out following the code of ethics of the American Anthropological Association [32] and the International Society of Ethnobiology Code of Ethics [33]. Oral prior informed consent was acquired.

The listed taxa (Table 1) were identified using specimens collected by informants in the forest or in the village. During freelisting we separately asked which species of wild vegetables (including underground organs), wild fruits and wild mushrooms were used. Making three separate freelists enabled comparison of the use of these categories and helped elicit answers from the respondents, who categorized the studied wild products in a similar way [34, 35]. Freelists were made orally and written down on the spot by our team. The conversations were additionally recorded using a digital sound recorder. The interviews were carried out in the local Thebo Tibetan dialect with the use of a local translator, or in Chinese if the interviewee could fluently speak this language. Many older men and nearly all middle-aged and elder women speak only Tibetan, whereas teenagers and young people in their twenties learned Mandarin Chinese at school and could facilitate communication. In contrast to their parents and grandparents they could also write down the plant names using Tibetan script. We recorded the local Tibetan names and transcribed them according to the International Phonetic Alphabet, and sound recordings of plant names were deposited in the Digital Repository of the University of Rzeszów [36].

**Table 1:** The list of fungi and plants used for food in Zhagana valley

Latin name checked in the Plant List	IPA pronunciation of local name [etymology given in square brackets], pronunciations without clear sound recordings in () brackets	Part used	Preparation	No. of interviews where mentioned	Specimen Number for fungi it begins with WA 00000517; for plants begins with WUK Kang
Fungi					
*Agaricus campestris* L. (Agaricaceae)	po χá χa mbi	m	boiled / fried	13	14
*Gomphus clavatus* (Pers.) Gray (Gomphaceae)	tɕu tsə́ χɔ̃ [square mushroom]	m	boiled / fried	10	24
*Carathelasma imperiale* (Quél.) Singer (Tricholomataceae)	χɔ̃ bo du	m	boiled / fried	11	37
*Lactarius deliciosus* (L.) Gray var. *deterrimus* (Russulaceae)	χɔ̃ má, χɔ̃ má má [red mushroom]	m	boiled / fried, dried for winter, highly valued	23	25, 32
*Leucopaxillus giganteus* (Sowerby) Singer (Tricholomataceae)	χɔ̃ kǎː [white mushroom]	m	boiled / fried	11	13, 15
*Clitocybe fragrans* (With.) P. Kumm. (Tricholomataceae) *Russula aurea* Pers., *Russula formula* Jul. Schäff. *Russula* sp. (at least 3 species of *Russula* are used) (Russulaceae)	si di χõ [bird mushroom]	m	boiled / fried, Russula is eaten raw	3	*C. fragrans* – 26, *R. formula* – 19, 35, *R, aurea* – 33, *R.* sp. – 27, 38
an edible lichen	wɔ tsə́ lɯɣ	m		1	–
*Morchella elata* Fr. (Morchellaceae)	kú ku χɔ̃ [cuckoo mushroom]	m	boiled / fried, dried for winter, mainly for sale, highly valued	11	10
*Auricularia auricula judae* (Bull.) Quél. (Auriculariaceae)	duː na ʑʉ (na ju) [du = tree, naju = ear]	m	boiled / fried, dried for winter	10	17
*Ramaria* spp. (Gomphaceae)	sa sə́ si sʉ [*sasa* are white forms, *sisi* – yellow and *mama* – red ones, red is best, white the worst]	m	boiled / fried, highly valued	16	12, 16, 18, 31, 36
*Sarcodon* sp. (*S.* cf *scabripes* (Peck) Banker?) (Bankeraceae)	lá χɔ̃ [eagle mushroom]	m	boiled / fried	15	32
unidentified fugus	ta pu ta χṍ	m	boiled / fried	9	–
unidentified fugus	(po dzɨ h χõ)	m	boiled / fried	3	–
unidentified fugus	si χõ	m	boiled / fried	3	–
unidentified fugus	(χõ dõ ʐu)	m	boiled / fried	10	–
unidentified fungus	ɕa χõ	m	boiled / fried	2	–
unidentified fungus	ni mbi χõ	m	boiled / fried	3	–
unidentified fungus	tɕo χõ	m	boiled / fried	1	–
unidentified fungus	dʐa si χõ	m	boiled / fried	4	–
unidentified fungus	(χõ tuo ʑaoʑa)	m	boiled / fried	1	–
Vascular plants					
*Allium chrysanthum* Regel (Alliaceae)	ndzy ri, gu sɪ	fl, l	spice, dried for winter, highly valued	22	32
*Allium cyaneum* Regel (Alliaceae)	dzǎː	fl, l	spice, dried for winter, highly valued	18	37
*Allium* sp. (Alliaceae)		fl, l	spice	1	–
*Allium* sp. (white flowers) (Alliaceae)	tɕʰa lá gu du	fl, l	spice, dried for winter, highly valued	15	45
*Angelica* cf *laxifoliata* Diels (Apiaceae)	ŋo tɕo lá	l	boiled / fried	4	38
*Aralia chinensis* L. (Araliaceae)	ka mó li ta	l (buds)	boiled / fried	1	26
*Artemisia* sp. (Compositae)	mo ta χa	l	boiled / fried		–
*Berberis circumserrata* (C.K.Schneid.) C.K.Schneid. (Berberidaceae)	tsʰa ma si tsʰən, si tɕʰã́	l, fl	raw snack	1	18
*Berberis potaninii* Maxim. (Berberidaceae)	tsʰa ma si tsʰən, si tɕʰã́	l, fl	raw snack	1	21
*Berberis* sp. (Berberidaceae)	tsʰa ma si tsʰən, si tɕʰã́	l, fl	raw snack	1	36
*Capsella bursa-pastoris* (L.) Medik. (Brassicaceae)	sʰo ká	L	boiled / fried	1	40
*Carum buriaticum* Turcz. (Apiaceae)	dəm ^b^ gu ɲi	F	powdered, used as sausage spice	11	24
*Chenopodium album* L. (Amaranthaceea)	ni lɔ́	L	boiled / fried	15	10
*Cirsium* sp. (Asteraceae)	tʰo xá	L	boiled / fried	2	39
*Elaeagnus rhamnoides* (L.) A.Nelson (Eleagnaceae)	la tá	F	raw snack	10	11
*Epilobium angustifolium* L. (Onagraceae)	ŋo tɕo la	Sh	boiled / fried, only famine food	1	33
*Equisetum* sp.? (Equisetaceae)	na dza ko rẽ	Sh	boiled / fried	3	–
*Fragaria orientalis* Losinsk. (Rosaceae)	a ji sa	F	raw snack	21	27
a Gentianaceae species	a nə́ ne, me tu pa pa	N	raw snack	5	–
*Lonicera* sp. (Caprofoliaceae)	ra nə́	F	raw snack	12	–
*Ixeris chinensis* (Thunb. ex Thunb.) Nakai (Asteraceae)	pʰa ki	L	boiled / fried	7	30
*Mentha canadensis* L. (Lamiaceae)	(dəm ^b^ ja na)	L	boiled / fried	1	25
*Notopterygium incisum* K.C.Ting ex H.T.Chang (Apiaceae)	ŋɔ́ tɔ	L	boiled / fried, dried for winter, highly valued	23	35
*Oxytropis* sp. (Fabaceae)	rə ŋge ɕe li	F	famine food	1	41
*Persicaria vivipara* (L.) Ronse Decr. (Polygonaceae)	ræ mbə́	F	seeds soaked overnight to remove bitterness	20	12
*Picea asperata* Mast. (Pinaceae)	tɔ tɕʰə́	Ss	raw snack		44
*Picea wilsonii* Mast. (Pinaceae)	tɔ tɕʰə́	Ss	raw snack		43
*Potentilla anserina* L. (Rosaceae)	tsɔ̃̌	R	boiled with butter and sugar/rice to make a ceremonial dish, highly valued	17	16
*Prinsepia uniflora* Batalin (Rosaceae)	ɔsʰi	F	raw snack	2	42
*Prunus salicifolia* Kunth (Rosaceae)	tɕe lə́	F	raw snack	11	34
*Pteridium aquilinum* (L.) Kuhn var. *latiusculum* (Desv.) Underw. ex A. Heller. (Dennstaedtiaceae)	ɕá la	L	boiled / fried, dried for winter, highly valued	30	6
*Rheum officinale* Baill. (Polygonaceae)	lají	St	raw snack on mountain treks	14	29
*Rheum palmatum* L. (Polygonaceae)	tɕĩ́, zɔ zẽ́	St	raw snack on mountain treks	13	19
*Ribes alpestre* Wall. ex Decne. (Grossulariaceae)	sʰe ró	F	raw snack	17	9
*Ribes vilmorinii* Jancz. (Grossulariaceae)	sʰi nã́	F	raw snack	13	7
*Rosa omeiensis* Rolfe (Rosaceae)	sa ka də la zə́	Sh	raw snack	8	28
*Rubus amabilis* Focke (Rosaceae)	sʰi mã́	F	raw snack	18	8
*Rubus pileatus* Focke (Rosaceae)	sʰi mã́	F	raw snack	x	2
*Rubus pungens* Cambess (Rosaceae).	sʰi mã́	F	raw snack	x	3
*Rubus xanthocarpus* Bureau & Franch. (Rosaceae)	sʰĩ	F	raw snack	15	4
*Rumex acetosa* L. (Polygonaceae)	a rá sa mbu	L	raw snack	13	14
*Salix* spp. (Salicaceae)	tɕã́ lǎ	Fl	?	3	–
*Salvia przewalskii* Maxim. (Lamiaceae)	də mbə ra ´nə, a ne nə nə [goat nipples]	N	raw snack	12	17
*Sinopodophyllum hexandrum* (Royle) T.S.Ying (Berberidaceae)	a mɛ̃ pʰa [toy pig]	F	raw snack	11	22
*Sonchus arvensis* L. (Asteraceae)	pʰa ki [pig grass]	L	boiled / fried	7	46
*Sorbus koehneana* C.K.Schneid (Rosaceae)	dʐə mə ga ɕí	F	raw snack	11	20
*Sorbus tianschanica* Rupr. (Rosaceae)	dʐə mə ga ɕí	F	raw snack	11	1
*Stachys affinis* Bunge. (Lamiaceae)	kʰa tə ´tsɔːwá	R	tubers boiled in the past	2	15
*Stellera chamaejasme* L. (Lamiaceae)	ra rə tɕá	R	famine food in the past	1	31
*Thlaspi arvense* L. (Brassicaceae)	dəmbə tʂǎ	L	seeds pressed for temple oil	14	5
*Triosteum pinnatifidum* Maxim. (Caprifoliaceae)	rə gu ɲi dɔ	F	raw snack	4	23
*Urtica dioica* L. (Urticaceae)	sá tsə	L	boiled / fried, also for dumpling filling and as cure for swollen legs	15	13
unidentified plant from grasslands	ŋo rə ba	L	boiled / fried	5	–
unidentified plant from grasslands	a ló ji lo	L	boiled / fried	4	–

*f* fruits, *fl* flowers, *l* leaves or buds, *m* mushroom, *n* nectar sucked, *r* roots, rhizomes, tubers or bulbs, *sh* asparagus-like young shoots, *ss* solidified sap (resin), *st* peeled stalks

Voucher specimens of fungi were deposited in the herbarium of the University of Warsaw (WA), and plants were deposited in the Herbarium of the Northwest A&F University in Yangling (WUK). Plants were identified using the standard identification key concerning local floras, and their names follow the Plant List [37]. Fungi names follow the Index Fungorum [38].

Around half of the fungi specimens (those whose specimens were gathered) were successfully identified using the DNA barcoding technique [39, 40]. Fungal DNA was extracted from a small part of each fruiting body using a Plant and Fungi DNA Purification Kit (Eurx), following standard protocol. The PCR cocktail was composed of 4 ml DNA extract, 0.5 ml each of the primers (ITS5 and ITS4 in 10 nmol concentration) and 5 ml Type-it Microsatellite PCR Kit (Qiagen). PCR was performed using the following thermocycling conditions: an initial 15 min at 95 °C, followed by 35 cycles at 95 °C for 30 s, 55 °C for 30 s, 72 °C for 1 min, and a final cycle of 10 min at 72 °C. PCR products were estimated by running 5 ml DNA amplicon on 1.5 % agarose gel for 30 min. The PCR products were sequenced with the use of ITS4 primers at the Laboratory of Molecular Biology of Adam Mickiewicz University (Poznań). The obtained sequences were verified visually on chromatograms using BIOEDIT. Nuclear ITS sequences obtained in this study are deposited in GenBank [41], with the accession numbers listed in Table 2.

**Table 2:** The results of DNA barcoding

Molecular identification	Accession number	Best match sequence / accession number	E-value	Sequence Similarity (%)
Agaricus campestris	KX008974	Agaricus campestris FJ223223	0.0	98
Ramaria sp.2	KX008975	Ramaria sp. UDB024083	0.0	97
Lactarius deliciosus var. deterrimus	KX008976	Lactarius deliciosus EF685060	0.0	99
Lactarius deliciosus var. deterrimus	KX008977	Lactarius deliciosus EF685060	0.0	99
Russula sp.	KX008978	Russula sp. EU057100	0.0	98
Auricularia auricula-judae	KX008979	Auricularia auricula-judae FJ478123	0.0	100
Morchella elata	KX008980	Morchella elata GQ249054	0.0	100
Sarcodon sp.	KX008981	Sarcodon scabripes JN135191	0.0	96
Leucopaxillus giganteus (Sowerby) Singer	KX008982	Leucopaxillus giganteus JQ639151	0.0	98
Leucopaxillus giganteus (Sowerby) Singer	KX008983	Leucopaxillus giganteus JQ639151	0.0	99
Ramaria sp. 3	KX008984	Ramaria cf coulterae JX310420	0.0	94
Russula firmula	KX008985	Russula firmula KJ867373	0.0	99
Russula sp.	KX008986	Russula sp. LC035243	0.0	99
Catathelasma imperiale	KX008987	Catathelasma imperiale UDB007898	0.0	97
Gomphus clavatus	KX008988	Gomphus clavatus UDB011658	0.0	99
Ramaria sp.1	KX008989	Ramaria sp. UDB013130	0.0	94
Ramaria sp.1	KX008990	Ramaria sp. UDB013130	0.0	94
Ramaria sp1	KX008991	Ramaria sp. UDB013130	0.0	94
Clitocybe fragrans	KX008992	Clitocybe fragrans UDB021263	0.0	99

## Results

**Fig. 4:**
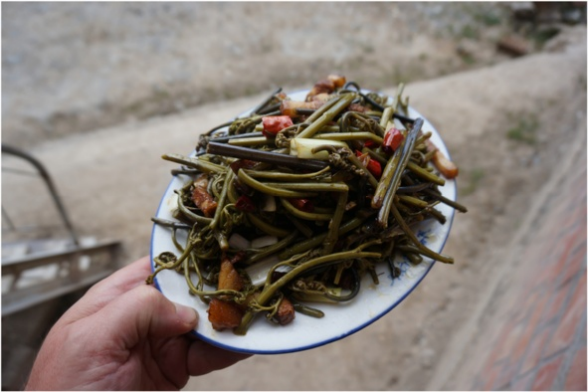
Fried *Pteridium* fronds. The fronds were dried and reconstituted in water before frying

**Fig. 5:**
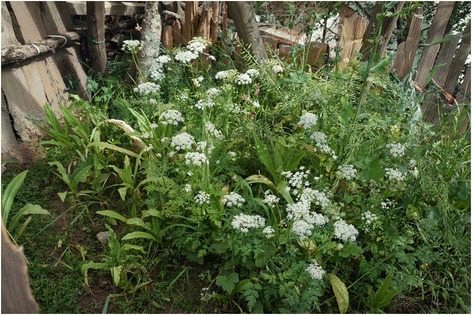
*Nothopterigium* is sometimes planted in home gardens as it is appreciated for its celery-like fragrance

**Fig. 6:**
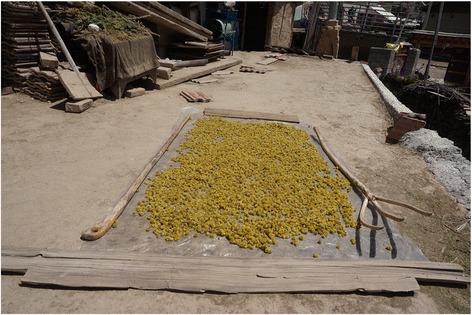
*Allium chrysanthum* flowers dried in the sun for winter

**Fig. 7:**
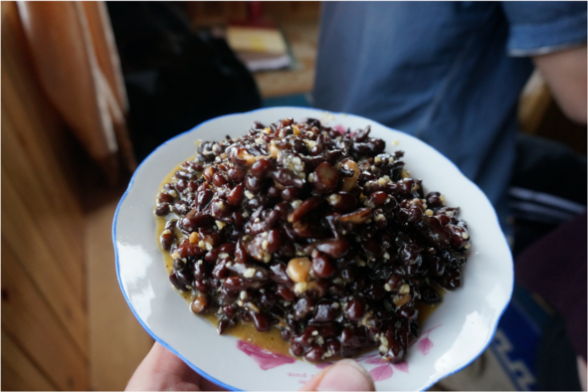
*Potentilla anserina* tubers boiled and served with butter and sugar. This is an expensive traditional dish served on important holidays

**Fig. 8:**
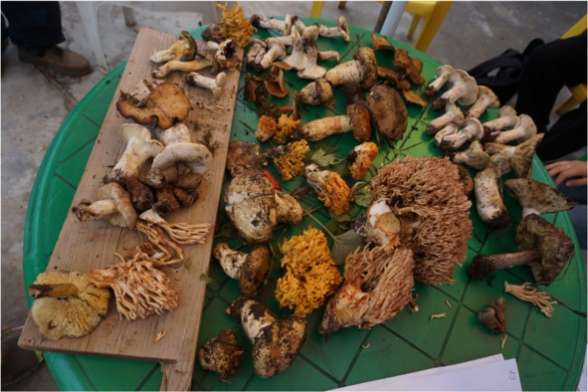
A collection of edible and non-edible mushrooms ready for sorting

Among the twelve most frequently mentioned wild foods are five species of wild vegetables – *Pteridium aquilinum* (L.) Kuhn (Fig. 4), *Notopterygium incisum* K.C.Ting ex H.T.Chang (Fig. 5), *Allium chrysanthum* Regel (Fig. 6), *A. cyaneum* Regel and *Chenopodium album* L., two taxa of fungi *Lactarius deliciosus* and *Ramaria* spp. (Fig. 7), three species of fleshy fruits (*Fragaria orientalis* Losinsk, *Rubus* spp., *Ribes alpestre* Wall. ex Decne) and two species used as staple foods (*Persicaria vivipara* (L.) Ronse Decr. syn. *Polygonum viviparum* L., edible seeds) and *Potentilla anserina* L. (edible tubers, Fig. 8).

We recorded the use of 54 species of vascular plants (Table 1). We also recorded the use of 22 mushroom taxa (Tables 1 and 2), which formed the largest category of wild foods. Fruits formed the largest category of food plants, with 21 species, larger than the wild greens category, which included 20 species eaten after boiling or frying and 7 as raw snacks. We also recorded the alimentary use of 10 species of edible flowers and 3 species with underground edible organs. On average, 20.8 edible taxa were listed per interview (median – 21). The most-listed category of wild foods was green vegetables (mean – 7.5 species, median – 8 species), but fruits and mushrooms were listed nearly as frequently (mean – 6.3, median – 6 and mean – 5.8, median – 6 respectively). Other category lists were very short: flowers (mean – 1.3, median – 1), underground edible parts (mean – 0.7, median – 1).

Most wild vegetables and mushrooms are usually boiled, sprinkled with hot oil and served as side-dishes. Wild fleshy fruits are collected mainly by children and eaten raw. Some green parts of plants are eaten as raw snacks: plants with a sour taste (*Rumex* leaves*, Rheum* peeled stalks), solidified spruce sap and nectar sucked out of flowers.

In times of famine or grain scarcity *Persicaria vivipara* fruits were mixed with barley and used to make flour. This was practiced even up until the 1980s. Other wild staples are the small tubers of *Potentilla anserina.* They are still gathered now, but are treated only as ceremonial foods, being served during New Year celebrations, funerals and other ceremonial occasions. Their rarer use stems from a very tedious gathering procedure. The tubers are dug out by women in late autumn or early spring. One woman can gather 0.5–1 kg of tubers per day. In the past they also constituted emergency food. Several informants observed changes in the frequency with which wild foods are collected: adults collect and eat less wild vegetables and children snack less on wild fruits. Most people usually use only a few wild vegetables, such as *Allium* spp., *Pteridium* and *Notopterygium.* Some people have stopped eating *Chenopodium* and *Urtica.* Due to the increasing involvement of tourism in the valley in the last 5 years, people do not have time to gather fungi in summer, at the peak of the tourist season.

**Fig. 9:**
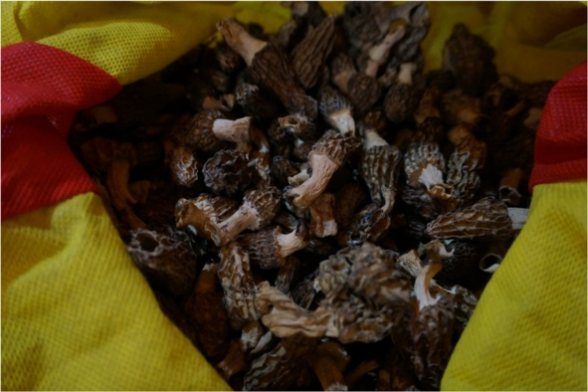
Dried *Morchella elata* for winter use or for sale

Practically all families dry wild vegetables for later use, however they do not lacto-ferment them. People usually dry bracken (*Pteridium*) fronds*, Nothopterigium* leaves and wild garlic (*Allium*) flower heads. They also dry a few species of mushrooms, mainly morels (*Morchella conica*; Fig. 9) and milk velvet caps (*Lactarius deliciosus* var. *deterrimus*). Morels are an important article of commerce, as is the medicinal *Cordyceps sinensis* mushroom, which was regarded by our informants as medicinal and not an edible mushroom. Some of our informants stored a few large sacks of morels for sale.

## Discussion

The few plant names available in the only other existing paper dealing with Thebo vocabulary [25] are very close to those recorded during the field trip, for instance *Potentilla anserina* (tsõ^L^ in [25], tsõ^L^ in the recordings, from Old Tibetan *gro.ma*) and the word for mushroom (x^h^awũ in [25], χõ in the recordings, from Old Tibetan ɕa.mo). Note that the sound change from Old Tibetan gr- to Thebo ts- in the word for *Potentilla anserina* is unique, among the varieties spoken in this region, to the Thebo language (Chone Tibetan for instance has tɕũ:^L^wa:^H^, [42]).

The number of wild taxa eaten in the studied valley is relatively low, much lower than in the lower elevations and in the Qinling Mountains. However the main reason for this is the lower number of species available in a landscape dominated by nearly pure spruce forests. The average length of freelisted wild foods is quite long and similar to other places in the mountains of western-central China. On the other hand, a particularly large number of fungi are eaten, and in this case, the spruce forests are a favourable habitat for many mycorrhizal edible taxa.

Interestingly, the composition of the list of edible taxa highly resembles many areas of Eastern Europe [43–50]. The following similarities may be noted:

- the large number of fungi taxa consumed,
- similar genera or even species eaten (e.g., *Rumex acetosa, Urtica dioica, Chenopodium album, Lactarius deliciosus, Agaricus campestris, Fragaria, Rubus, Sorbus, Ribes, Agaricus, Russula* and *Morchella*),
- similar length of lists of fungi, fruits and wild vegetables consumed,
- a high appreciation of the sour taste, in contrast e.g., to Chinese people in the Qinling Mts. [7, 8].

**Fig. 10:**
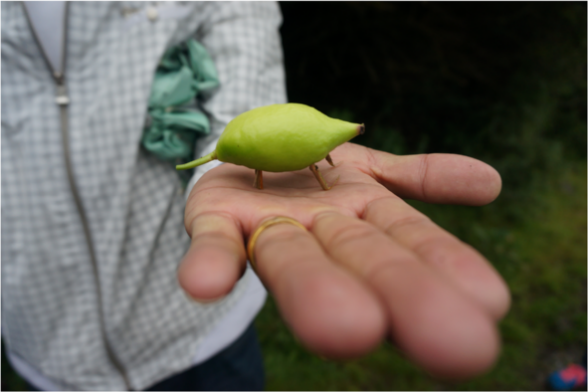
*Sinopodophyllum hexandrum* is called ‘toy pig’ as apart from being eaten it is used for making miniature pigs, as children’s toys

However, the list of the collected taxa and their processing also displays features typical for other east Asian communities – e.g., the use of *Pteridium aquilinum,* drying wild vegetables for winter, a lack of preserves made of fruits and fungi. The list of species is quite similar to the edible plants used by Tibetan communities recorded by Boesi in the vicinity of Litang (Sichuan) [1]. The people in Litang also eat *Potentilla* roots, *Allium, Rosa* shoots, *Berberis* flowers, *Rheum* stalks, *Sinopodophyllum* fruits (Fig. 10), *Urtica, Chenopodium* and *Thlaspi* shoots, food is spiced with *Carum* fruits etc. and *Persicaria vivipara* is used there under a nearly identical name (*rambu*). A very similar composition of wild foods was also recorded in south-central Tibet in another study [4]. There, *Rheum* spp. stalks, *P. anserina* bulbs, *Chenopodium album, Allium* and *Urtica* are also eaten.

It should be emphasized that although edible mushrooms are highly appreciated in China, it is only in Yunnan that larger numbers of mushroom species are used for food (e.g., [51, 52]). In the areas east of our study area, at lower elevations, in Gansu and Shaanxi, we recorded much lower numbers of species used and the very rare occurrence of drying mushrooms for winter use. For the moment, the use of over 20 fungi taxa in a wild valley makes the studied communities the most mycophilous places outside Yunnan. However this may simply result from the low number of ethnomycological studies in central Asia. For example it is worth noting that the number of edible fungi taxa recorded in this study (22) is similar to that used by Sherpa people in Nepal, on the other edge of the Tibetan Plateau (26 species) [53].

## Conclusions

The studied community uses a nearly equal number of fungi, fruit and wild vegetable species. The list of taxa used is not very long for a rural mountain community in Asia, but this stems mainly from the low species richness of the vegetation surrounding the villages.

The ZhaganaTibetans are probably the most mycophilous ethnic group in China outside Yunnan.

The composition of wild food plant taxa is typical for Tibetan speaking areas (e.g., the use of rhubarb shoots, *Potentilla anserina, Persicaria vivipara*).
